# Performance of Self-Sensing Cement-Stabilized Sand under Various Loading Conditions

**DOI:** 10.3390/s24061737

**Published:** 2024-03-07

**Authors:** Mohammad Jawed Roshan, Mohammadmahdi Abedi, António Gomes Correia, Raul Fangueiro

**Affiliations:** 1 Department of Civil Engineering, ISISE, ARISE, University of Minho, Campus de Azurém, 4800-058 Guimarães, Portugalagc@civil.uminho.pt (A.G.C.); 2 Department of Textile Engineering, Centre for Textile Science and Technology, University of Minho, Campus de Azurém, 4800-058 Guimarães, Portugal; rfangueiro@dem.uminho.pt

**Keywords:** piezoresistive performance, loading condition, self-sensing, cementitious composite

## Abstract

Numerous elements, such as the composition and characteristics of carbon nanomaterials, the composition and characteristics of the matrix material, moisture levels, temperature, and loading circumstances, influence the piezoresistive behavior of self-sensing cementitious composites. While some past research has explored the impact of some of these factors on the performance of self-sensing cementitious composites, additional investigations need to be conducted to delve into how loading conditions affect the sensitivity of self-sensing cement-stabilized composites. Therefore, this study explores the influences of various loading conditions (i.e., location of loading regarding the location of recording electrodes, and loading level) on the electromechanical performance of self-sensing cement-stabilized sand. To this end, firstly, the evaluation of the percolation threshold based on 10% cement-stabilized sand specimens containing various multiwall carbon nanotubes (MWCNTs) and graphene nanoplatelets (GNPs) was performed. Then, 10% cement-stabilized sand containing 4% MWCNTs/GNPs was tested under various cyclic compressive stresses. The results suggested that the distance between the loading area and the electrode location used for recording the electrical resistance significantly impacted the sensitivity of cement-stabilized sand. Optimal sensitivity was achieved when the electrodes were positioned directly beneath the loading area. Moreover, the study showed that the stress sensitivity of self-sensing cement-stabilized sand increased proportionally with the stress level. An examination through scanning electron microscopy (SEM) demonstrated that the loading condition influences the bridging characteristics of carbon nanomaterials in cement-stabilized sand, leading to diverse electromechanical behaviors emerging based on the loading condition. This study underscores the importance of considering specific parameters when designing self-sensing cement-stabilized sand for application in practical field use.

## 1. Introduction

Structural failures, caused by different factors including environmental factors, operational conditions, and extensive loading, result in severe economic losses and safety risks. Although various soil improvement techniques are utilized, the failure of geotechnical infrastructure, even when in an improved condition, is also reported [1,2,3]. Therefore, the continual monitoring of civil engineering infrastructure is essential to increasing service life and enhancing safety through the early detection and identification of detrimental conditions. To this end, structural health monitoring (SHM) is employed through diverse systems for the real-time assessment of civil infrastructure conditions. Among other systems, self-sensing cement-based materials are widely used as SHM systems to detect degradation, cracks, and damage under stress/strain in civil engineering infrastructure. The wide usage of multifunctional cementitious composites is attributed to their advantages, such as excellent mechanical and durability properties, low cost, high gauge factor, and considerable compatibility with civil engineering elements [4]. In addition, conventional methods can provide local surficial information about structural conditions, while self-sensing cementitious composites enable the overall integrity of structures; hence, this is a considerable advantage.

To establish the sensing capability, various conductive fillers, including steel fibers [5,6,7,8], micro- and nanocarbon fibers [9,10,11,12], carbon black structures [13,14,15,16], carbon nanotubes [17,18,19,20,21], graphene nanoplatelets [22,23,24,25,26,27], steel fibers [28,29], and hybrid conductive fillers [30,31,32,33], are incorporated into cementitious composites. However, carbon-based functional materials vastly increase cementitious composite piezoresistivity and enhance mechanical characteristics [34,35]. The change in electrical resistance under induced stress and strain provides the concept of structural health monitoring (SHM) when applying the self-sensing cementitious concept. The performance level of self-sensing cementitious composites in terms of self-sensing capability depends on diverse factors, including the type of matrix material used (i.e., nonconductive part), water content, binder types, electrode type, electrode configuration, the type of conductive fillers, the surface condition of conductive fillers, the percentage of conductive fillers, the dispersion quality, the type of electrical circuit, and loading conditions. For instance, the self-sensing cement mortar exhibits better piezoresistive performance than the self-sensing concrete due to the existence of small pores between particles. In this context, D’Alessandro et al. [36] observed a rise in the signal-to-noise ratio of electrical resistance when transitioning from concrete to cement paste, confirming the enhancement of piezoresistive capability in these composites as the quantity and dimensions of matrix materials decrease. Moreover, Parvaneh and Khaibani [19] noted the superior performance of self-sensing cement paste compared to self-sensing mortar and concrete.

The micropore condition will affect the resistivity of self-sensing cementitious composites as well. In this regard, Liu et al. [31] reported an increase in electrical resistivity with decreasing moisture content. This issue can be attributed to the emergence of insulated space in the micropores due to decreasing moisture content. To lower the emergence of micropores during casting, Choi et al. [37] employed various concentrations of defoamer for CNT cementitious composites. Their findings revealed decreased electrical resistance with increasing defoamer concentration due to suppressed voids. The agglomeration of CNTs is another factor that causes porosity in cementitious composites, thus resulting in decreased piezoresistive performance and mechanical strength. To tackle this challenge, the use of silica fume in self-sensing cementitious composites was investigated [34]. The subsequent findings revealed the increasing dispersion of CNTs in cementitious composites with increasing silica fumes, thus resulting in increased piezoresistivity and mechanical strength with increasing silica content. Furthermore, in order to attain a stable and reliable piezoresistive performance in cementitious composites, Wang et al. [38] developed a self-sensing cementitious composite employing hybrid conductive fillers (NGPs/CNTs/NCBs). Their results demonstrated a notably stable and high-performing piezoresistive capability under both monotonic and cyclic loading conditions. In a separate investigation [39], carbon nanotubes (CNTs) were directly synthesized on carbon fibers (CFs) to alleviate agglomeration issues, subsequently enhancing the mechanical and electromechanical performance of self-sensing cementitious composites.

The influence of various factors on the piezoresistive performance of self-sensing cementitious composites has been investigated in previous studies [40,41,42,43,44,45]. Yıldırım et al. [41] explored the influence of curing time and loading conditions (i.e., four-point bending, tensile, and uniaxial compression). Zhan et al. [42] investigated the influence of conductive filler concentration on piezoresistive performance. Meng et al. [46] evaluated the effects of conductive filler types and aging on the piezoresistive performance of self-sensing cementitious composites. The sensing capability of self-sensing cementitious composites in sensor, coating, and bulk forms was investigated under diverse loading conditions [47,48,49,50]. However, in most previous studies, compressive cyclic and monotonic loadings were applied directly on top of electrodes [14,49,51,52], which differed from some of the real scenarios in civil engineering infrastructure, in which loading may not be directly applied on top of electrodes. Previous research studies only evaluated the influence of the loading type (i.e., compressive, tensile, bending, cyclic, and monotonic) on piezoresistive performance [11,41,44,46,53,54]. Therefore, although the sensing capability of self-sensing cementitious composites is undeniable, the effects of loading conditions (i.e., location of loading regarding the location of recording electrodes and loading level) on the sensing capability of cementitious composites still need to be fully comprehended. In pursuit of this objective, while prior research has extensively explored alternative varieties of self-sensing cementitious composites (such as self-sensing cement paste, self-sensing mortar, and self-sensing concrete), the present investigation evaluates the efficacy of self-sensing cement-stabilized sand incorporating 4% MWCNT/GNP under diverse compressive cyclic loading scenarios. The development of this self-sensing, cement-stabilized sand, conducted within the framework of the In2Track3 project (a European-funded project), is intended for future application in the construction of transportation infrastructure layers. The hybrid MWCNT/GNP combination was employed to mitigate microporosity and consequently enhance the electromechanical performance of the self-sensing cement-stabilized sand, aiming to achieve consistent and reproducible electrical responses under loading conditions [55]. The electromechanical tests were conducted under various cyclic compressive loadings in order to assess the loading conditions in transportation infrastructure. The current study’s findings provide information regarding the effects of loading conditions (i.e., location of loading regarding the location of recording electrodes, and loading level) on the electromechanical properties of self-sensing cement-stabilized sand, which need to be considered before application in field projects. The findings of this research can be helpful in carefully arranging and configuring the electrodes used for electrical signal collection in a self-sensing cementitious composite system.

## 2. Material and Methods

### 2.1. Materials

**Figure 1:**
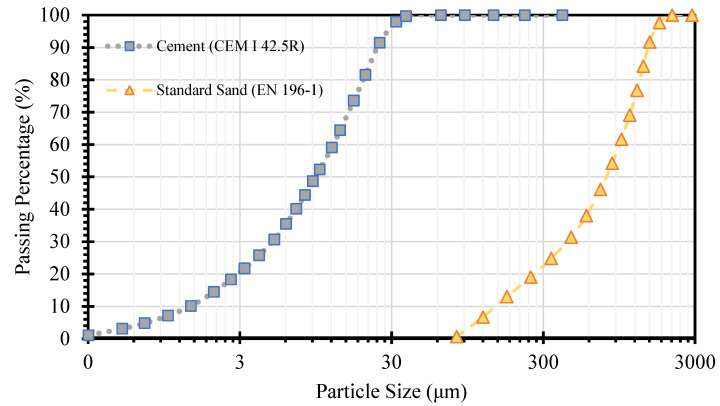
Grain size distribution of standard sand and ordinary Portland cement.

The relevant materials are categorized into matrix and conductive/functional materials in self-sensing cementitious composites. In the first step, the effects of adding various MWCNT/GNP concentrations on the impedance of cement-stabilized sand were evaluated using a PalmSens device. Based on the findings, further analyses were conducted to evaluate the effects of loading conditions on the piezoresistive performance of self-sensing cement-stabilized sand, although only on specimens containing 4% MWCNT/GNP. To stabilize the sand for being applied in transportation infrastructure sublayers, 10% ordinary Portland cement (OPC) was utilized. The porosity distribution is one of the main factors affecting the electromechanical characteristics of self-sensing cementitious composites [37]. Given this issue, standard sand was used in this study to minimize the effects of particle size and a nonhomogeneous distribution of porosity on the electromechanical properties of cement-stabilized sand. The grain size distribution (GSD) of ordinary Portland cement (OPC) and standard sand are depicted in Figure 1 according to EN 196-1, ISO 679: 2009, and EN 197/1-2011 standards [56,57]. Further details on the GSD and physical properties of the standard sand used in this study are tabulated in Table 1.

Depending on its composition, cement is categorized into over ten types [58]. However, in the current study, ordinary Portland cement (OPC) CEM 1, 42.5R was used as a binder agent due to its low cost, high effectiveness, considerable workability and density, progressive strength gain, and very high resistance to chemical reactions [59]. Given these features, this type of cement is commonly used to improve transportation infrastructure [60]. The chemical and physical properties of CEM 1, 42.5R employed in this study are presented in Table 2.

Diverse functional materials have been used in previous studies [61] to establish the sensing capability in cementitious composites. In the current study, hybrid carbon nanomaterials comprising MWCNTs and GNPs were utilized due to their remarkable synergistic effects on the mechanical and electromechanical characteristics of self-sensing cementitious composites [55]. The details of the MWCNTs and GNPs utilized in this study are tabulated in Table 3.

The agglomeration of carbon nanomaterials due to their massive specific surface area and energy is one of the main concerns in self-sensing cementitious composite fabrication. To tackle this challenge, dispersion techniques, including physical and chemical techniques, have been applied in previous studies [10,62]. In the current study, combined physical (i.e., sonication) and chemical (i.e., the addition of Pluronic F-127 into water) methods were employed to achieve the desired dispersion of MWCNT/GNP in water. In addition, to avoid foam formation due to the chemical reaction of the surfactant (Pluronic F-127), tributyl phosphate 97% was also used as a foam reducer.

### 2.2. Mixing Procedures and Sample Preparation

**Figure 2:**
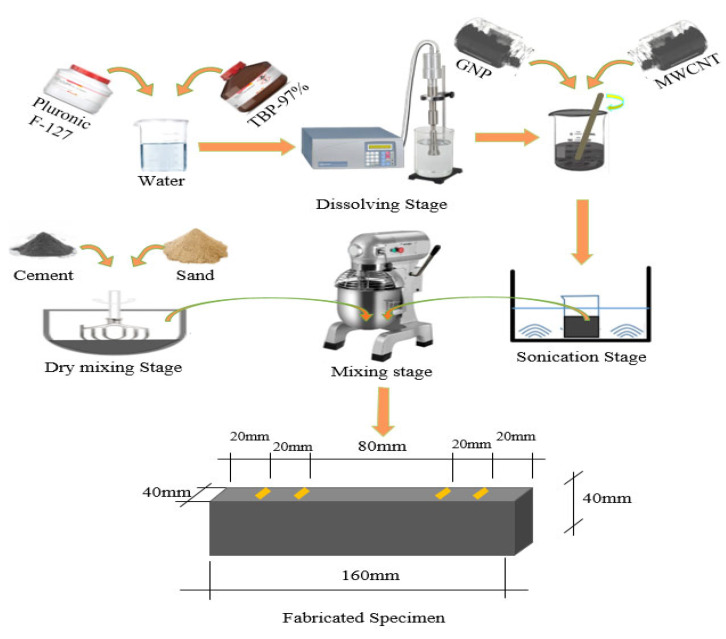
Mixing and sample fabrication stages.

Although the general fabrication procedures for self-sensing cementitious composites are similar to those of conventional cementitious composites, a few extra steps, including the dispersion of carbon nanomaterials and the installation of electrodes, are necessary for the fabrication of self-sensing cementitious composites. In the first step, therefore, we thoroughly dissolved 10% surfactant (Pluronic F-127) by weight of carbon nanomaterial and 50% TBP-97% by weight of surfactant in water (i.e., optimum moisture content). Then, we added 0.5%, 1%, 2%, 3%, and 4% MWCNT/GNP (1:1) by weight of dry sand to the obtained solution. After thoroughly stirring and mixing, bath sonication was employed to disperse the CNMs in the solution. It should be noted that the combined dispersion technique (i.e., using Pluronic F-127 and sonication) used in the current study was proven to be suitable for the dispersion of carbon nanomaterials in 2015 by Parveen et al. [63]. The dispersed CNMs were then added to the dry mixed sand and cement. The self-sensing cementitious composite components were thoroughly mixed in the mixer. Finally, samples with dimensions of 160 mm × 40 mm × 40 mm were fabricated according to the maximum dry density, which is usually considered for the compaction of the transportation layer. The prepared samples were tested after 28 days of curing in a humid room. The steps followed to mix the self-sensing cementitious composite and perform sample preparation are summarized in Figure 2. In brief, Table 4 illustrates the composition of components within the self-sensing cement-stabilized sand.

In prior investigations, combined conductive fillers were extensively employed to improve the mechanical and electrochemical properties of cementitious composites effectively [35,64,65,66]. The enhanced impact of hybrid conductive fillers on mechanical and electromechanical characteristics is attributed to their distinct physical attributes [67]. The combination of these conductive fillers, each with diverse physical properties, consequently leads to enhanced self-sensing functionality. Carbon nanotubes (CNTs), in comparison to other carbon derivatives, are costly, and achieving their proper dispersion presents challenges due to their high surface energy (resulting in significant van der Waals forces) and entanglement, leading to agglomeration [68]. Introducing graphene nanoplatelets (GNPs) into cementitious composites generates isotropic conditions owing to their two-dimensional structure [69], potentially resulting in uniform sensing capabilities in all directions. Because of their plate-like morphology, GNPs can enhance the load-carrying capacity of cementitious composites [70]. Consequently, the present investigation examines a combination of hybrid conductive fillers (MWCNTs/GNPs) in equal proportions (1:1) in order to employ their advantages equally. However, future studies should explore the impact of different proportions of MWCNTs/GNPs on the piezoresistive performance of self-sensing cement-stabilized sand.

### 2.3. Experimental Methods

**Figure 3:**
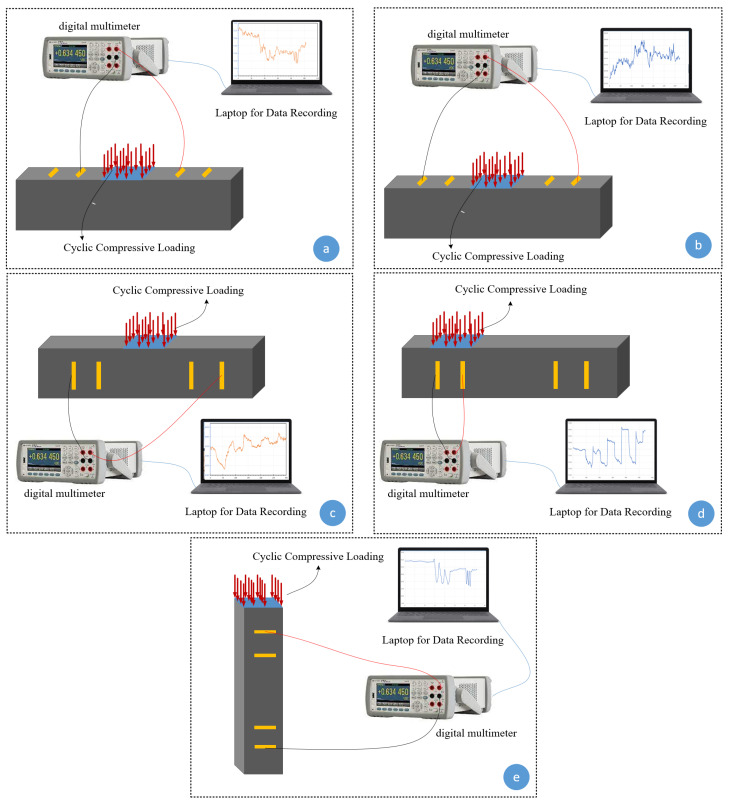
Various applied loading conditions (**a**–**e**) for piezoresistive performance measurement.

In the first step, the influence of adding various MWCNT/GNP on electrical impedance was evaluated using a PalmSens device (manufactured by PalmSens BV, Houten, The Netherlands). Then, further investigations were conducted on the specimens containing 4% MWCNT/GNP. In most previous studies, the electromechanical characteristics of self-sensing cementitious composites were investigated under the loading conditions shown in Figure 3e [23,71,72]. In this study, an electromechanical test was conducted under various compressive cyclic loading conditions to evaluate the sensing capability of the bulk self-sensing cementitious composite, as shown in Figure 3. The five scenarios shown in Figure 3 were considered for electromechanical testing. Using the loading conditions shown in Figure 3a,b, cyclic compressive loading was applied to the region between electrodes, and the electrical resistance was recorded from the inner and outer electrodes. In Figure 3c, cyclic compressive loading was exerted on the region between electrodes, and the electrical resistance was measured through the inner electrodes. The exact loading level and shape used in Figure 3c were executed on top of the electrodes in Figure 3d, and the electrical resistance was recorded from those electrodes under the loading region. In the last loading scenario, various compressive cyclic loading levels were applied to evaluate the effects of loading level on the piezoresistive performance of the self-sensing cementitious composite. To assess the influence of various compressive loading conditions on the piezoresistive behavior of self-sensing cement-stabilized sand, specimens were subjected to cyclic compression using a Lloyd 50 kN compressive loading machine. Concurrently, the electrical resistance was monitored during loading using a digital multimeter (Agilent 34461A 6½). It is important to note that the impact of loading conditions was assessed through the application of cyclic compressive loading within the elastic range of self-sensing cement-stabilized sand. Meanwhile, the influence of loading magnitude was investigated by subjecting the material to cyclic compressive loading, spanning from the elastic to the plastic regions.

In addition to the electromechanical test, microstructural characteristics were evaluated based on scanning electron microscopy (SEM) experiments in order to appraise the influence of MWCNT/GNP on the morphology of cement-stabilized sand. The specimens used for SEM analysis were prepared from the collapsed electromechanical testing sample. The SEM samples were coated with a Au–Pd thin film (30 nm) using a high-resolution sputter coater (Cressington 208HR, manufactured by Cressington Company, Watford, UK). Following the coating stage, the SEM experiment was conducted using 10 kV voltage and a secondary electron mode.

### 2.4. Piezoresistivity Measurements

**Figure 4:**
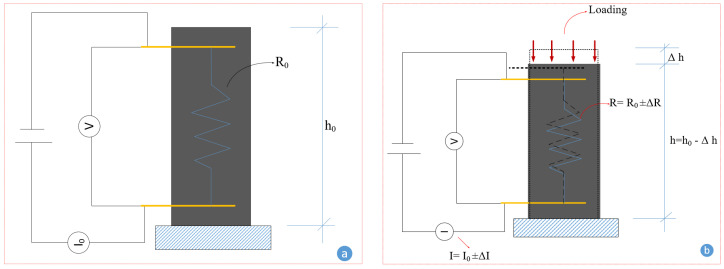
Electromechanical concept under compressive loading: (**a**) initial resistance, (**b**) resistance changes under loading.

The electrical resistance was recorded using an Agilent 34461A 6½ Digit digital multimeter (manufactured by Agilent Company, Santa Clara, CA, USA) during the loading scenarios mentioned above. The recording rate was adjusted to 10 times per second to thoroughly capture the electrical resistance under loading. Although the samples were cast with four electrode probes, a two-probe system with DC current was employed to record the electrical resistance in this study. The fractional changes in resistivity (FCR) were calculated according to Equation (1).

(1) $$FCR=\frac{∆R}{R_{0}}\approx \frac{∆\rho }{\rho_{0}}$$

where ∆R, ∆ρ, $R_{0}$, and $\rho_{0}$ are the fractional changes in resistance, fractional changes in resistivity, initial resistance, and initial resistivity, respectively. The changes in resistance after applying the load are schematically illustrated in Figure 4. Figure 4 shows that the resistance can increase or decrease depending on the integrity condition of the self-sensing cementitious composite under loading.

## 3. Results and Discussion

### 3.1. Influence of MWCNT/GNP Concentration on Electrical Impedance

**Figure 5:**
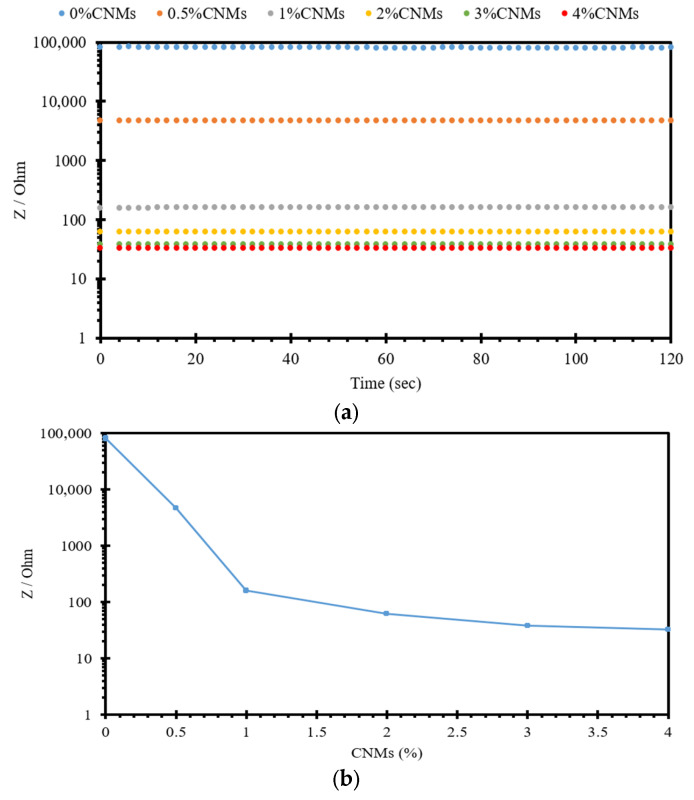
(**a**) Electrical impedance-time of self-sensing cement-stabilized sand containing various percentage of CNMs (MWCNT/GNP). (**b**) Electrical impedance–CNMs (MWCNT/GNP).

In the current study, a PalmSens device, which works based on AC, was employed to evaluate the influence of conductive fillers on the impedance of 10% cement-stabilized sand. Adding MWCNT/GNP in cement-stabilized sand produces random conductive pathways, resulting in decreased electrical impedance, as seen in Figure 5. Figure 5a illustrates the recorded electrical impedance over 120 s for specimens containing various MWCNT/GNP concentrations ranging from 0% to 4%. Regarding Figure 5a, it is seen that the electrical impedance decreases with increasing MWCNT/GNP concentrations. Figure 5b was plotted according to Figure 5a to evaluate the influence of MWCNT/GNP concentrations on the electrical impedance of 10% cement-stabilized sand, a construction material usually used in transportation infrastructure sublayers. Figure 5b indicates the drastic decrease in electrical impedance after adding 1% MWCNT/GNP, indicating the percolation threshold. The reduction rate in the electrical impedance of 10% cement-stabilized sand decreases with the increase in MWCNT/GNP to beyond 1%. This phenomenon is attributed to the concentration of forming conductive pathways originating from the addition of conductive fillers. In other words, the conductive pathways in 10% cement-stabilized sand will not be significant when the concentration of MWCNT/GNP is lower than 1%, resulting in a large electrical impedance. However, when the concentration of MWCNT/GNP is more than 1%, the electrical impedance is small due to the produced intensive conductive pathways within 10% cement-stabilized sand. Since the objective of this study is to evaluate the effects of loading conditions on piezoresistive performance of self-sensing cement-stabilized sand, 4% MWCNT/GNP was incorporated into 10% cement-stabilized specimens used for further analysis. This way, the highly sensitive cement-stabilized sand could be produced, minimizing the polarization effects during testing. Given the study’s objectives, a comprehensive cost analysis was not undertaken. However, to increase the applicability of the developed self-sensing cement-stabilized sand in practical projects, a thorough cost analysis will be essential in forthcoming research endeavors. Nonetheless, it should be acknowledged that, while the inclusion of MWCNTs/GNPs incurs additional costs compared to conventional cement-stabilized sand, potential savings from proper maintenance measures and prolonged service life facilitated by self-sensing cement stabilization can offset the expenses associated with MWCNTs/GNPs.

### 3.2. Effects of Distance of Loading Region from Electrodes on Piezoresistive Performance

**Figure 6:**
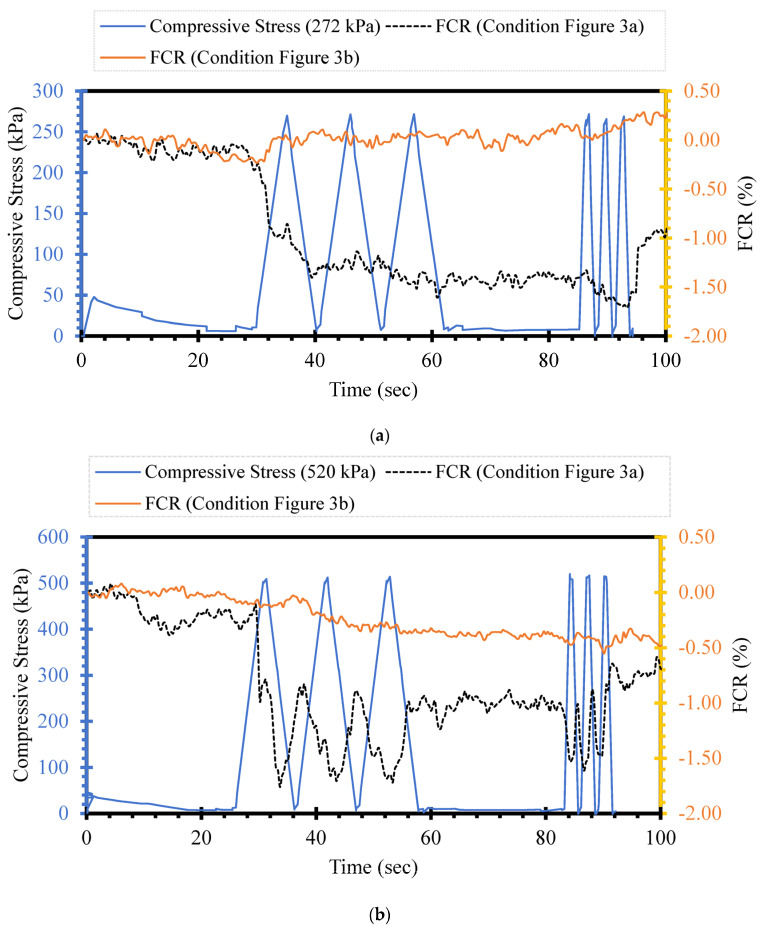

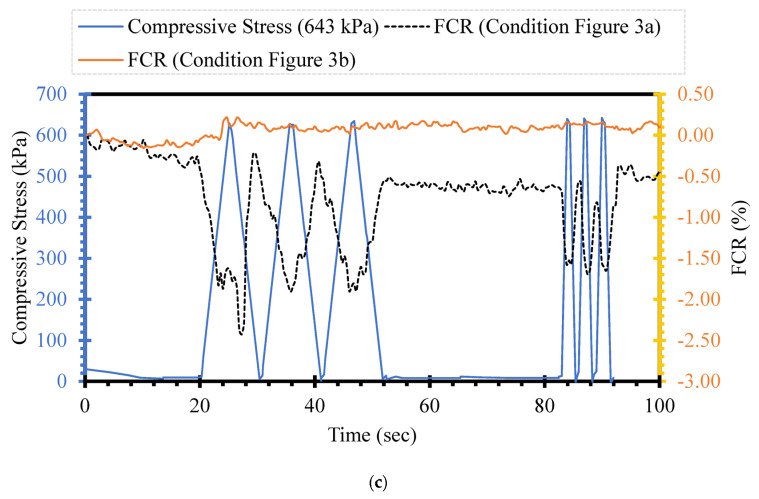
Compressive cyclic stress/FCR–time for loading conditions according to Figure 3a,b; (**a**) cyclic compressive stress = 272 kPa; (**b**) cyclic compressive stress = 520 kPa; (**c**) cyclic compressive stress = 643 kPa.

The piezoresistivity of self-sensing cementitious composites is the electrical resistance changes due to stress, strain, and damage. The stress-, strain-, and damage-sensing ability of self-sensing cementitious composites has been explored widely in previous studies [10,73,74,75]. However, in previous studies, loading was directly applied on top of electrodes [14,38,76,77]. Given this issue, this section discusses the influence of the loading region’s distance from the electrodes used for electrical resistance recording on the piezoresistive performance. Figure 6 illustrates the FCR changes for the loading conditions shown in Figure 3a,b. The blue line represents the cyclic compressive stress, and the black and yellow lines indicate the FCR changes for conditions a (close to the loading region) and b (far from the loading region), respectively, shown in Figure 3. Comparing the FCR changes in Figure 5 under the same stress level yields the conclusion that the loading distance from the electrodes used for electrical resistance recording significantly affects the piezoresistive performance of self-sensing cementitious composites. For instance, in Figure 6a, it is seen that the FCR changes under the loading condition of Figure 3a are evident compared to those in the loading condition of Figure 3b. The fluctuation in the piezoresistive behavior of self-sensing cement-stabilized sand in response to changes in the distance of loading from the recording electrodes can be attributed to the distribution of stress within the material body. An enhanced piezoresistive performance occurs when the recording electrodes are positioned within the stress distribution zone, while weaker performance is observed when the electrodes are located outside this region. To address this, various stress distribution techniques such as Bosinisque, Westergaard, multiple-layer analysis, vertical ratio (2:1), and vertical ratio (angle) can be considered for use on transportation infrastructure layers, allowing for the adjustment of electrode configuration accordingly [78].

Similarly, the differential response of FCR under compressive cyclic stress is obvious when examining the loading conditions shown from Figure 3a,b to Figure 6b,c. Therefore, it is concluded that the stress sensitivity of the self-sensing cementitious composite is affected by the loading distance from the electrodes used for electrical resistance recording. In addition, Figure 6 shows that stress sensitivity increases with increasing stress levels, rising from 272 kPa to 520 kPa and 643 kPa. However, the FCR changes with increasing stress level only become evident for the inner electrodes (condition a). The stress sensitivity for the outer electrodes does not appear under the maximum stress used in this part (643 kPa). Given the issues discussed, it is important to consider the effects of the loading region on the stress sensitivity of self-sensing cementitious composites before applying them in a real-world project. The electrode location employed to record electrical resistance plays a vital role in the stress sensitivity of self-sensing cement-stabilized sand.

### 3.3. Effects of Loading Position on Piezoresistive Performance

**Figure 7:**
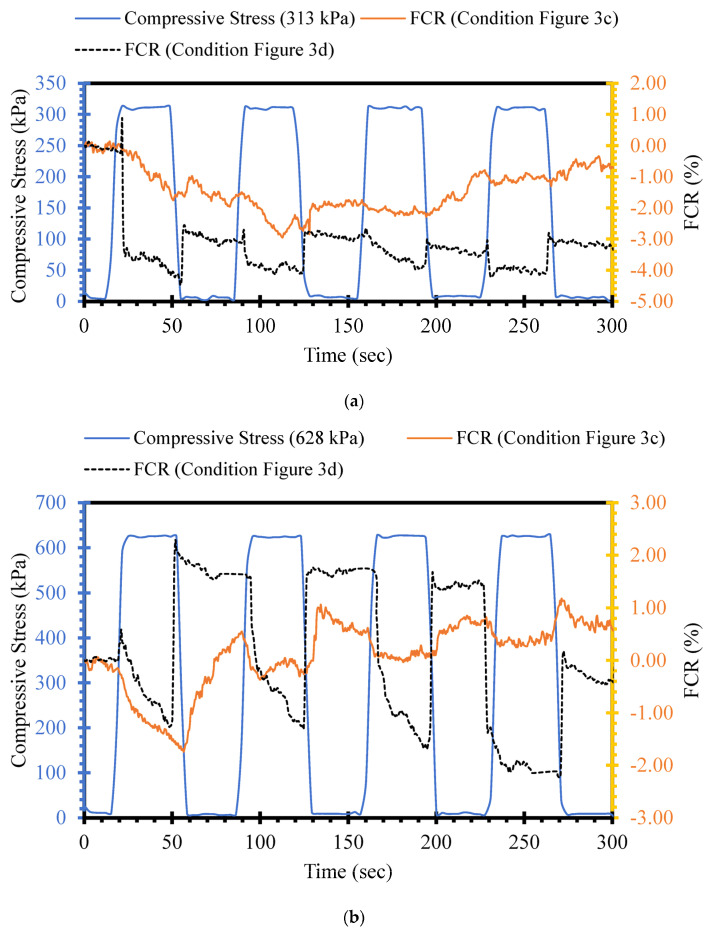

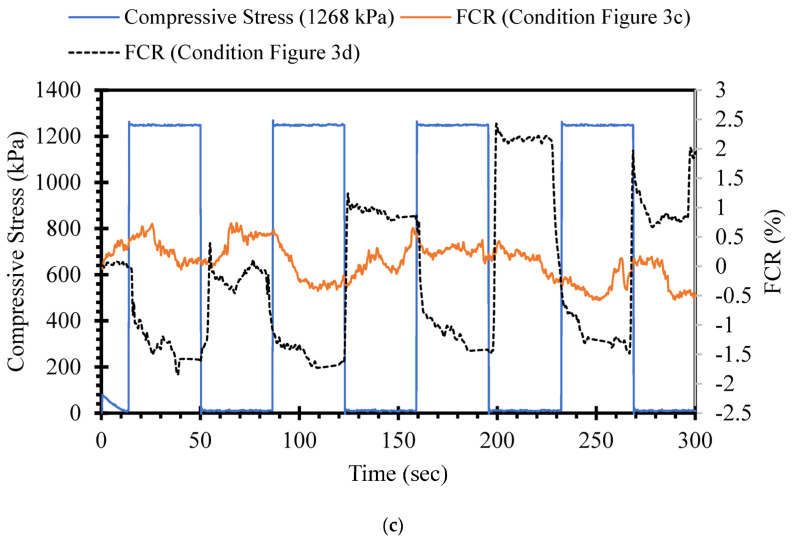
Compressive cyclic stress/FCR–time for loading conditions according to Figure 3c,d. (**a**) cyclic compressive stress = 313 kPa; (**b**) cyclic compressive stress = 628 kPa; (**c**) cyclic compressive stress = 1268 kPa.

To further evaluate the piezoresistive performance of self-sensing cement-stabilized sand with respect to loading conditions, three compressive cyclic stress levels (313 kPa, 628 kPa, and 1268 kPa) were applied to self-sensing cementitious samples, as shown in Figure 3c,d. Figure 7 depicts the FCR changes under cyclic stress for the loading conditions shown in Figure 3c (loading on the region between electrodes) and Figure 3d (loading on top of electrodes). In the case of the loading condition in Figure 3c (loading on the region between electrodes), in which the electrical resistance was recorded from the outer electrode, the FCR changes under compressive stress were not readily noticeable, as seen in Figure 7 in yellow. In contrast, the FCR trends obtained for the loading condition in Figure 3d (loading on the top of electrodes) followed the trend in compressive stress, as shown in black in Figure 7. This behavior is attributed to the conductive pathways that emerge under compressive loading. In the case of the loading condition in Figure 3c, the conductive pathways that emerge under loading are not contiguous, and electrical conduction disruption occurs between electrodes because they are situated far from the loading region. On the other hand, continuous conductive networks emerge between two electrodes under loading conditions, as shown in Figure 3d, since the loading area covers the region between electrodes. In other words, the covering of electrodes by the loading area causes strengthened conductive networks to form between two electrodes upon decreasing gaps between functional fillers, resulting in the increased stress/strain sensitivity of self-sensing cement-stabilized sand. The gauge factor, which indicates the strain sensitivity of sensors, is defined as the ratio of resistance change to strain change. For this reason, Poisson’s ratio is another factor that affects the sensitivity of piezoresistive sensors [79]. It is noteworthy that the gauge factor of metallic sensors can be directly correlated with Poisson’s ratio (ν) due to their very small resistivity compared to strain (∆ρρ<<∆LL) [80]. Conversely, the gauge factor of semiconductor materials like self-sensing cementitious composites, where resistivity variation is much larger than strain (∆ρρ>>∆LL ), depends on resistance changes over strain [81]. Consequently, the piezoresistive performance of self-sensing cementitious composites under loading conditions varies in different directions due to variations in Poisson’s ratio [82]. This can be explained by the fact that the variation in Poisson’s ratio of each component of self-sensing cement-stabilized sand under loading conditions significantly impacts piezoresistive performance. For example, differences in Poisson’s ratio between electrodes and self-sensing cement-stabilized sand cause interfacial stress, thus affecting the material’s piezoresistive performance, as discussed in a previous study on piezoelectric transducers [83]. In this context, it can be inferred that changes in the dimensions of electrodes depend on the loading conditions, resulting in different piezoresistive performances under various loads. Additionally, if self-sensing cement-stabilized sand is utilized in sensor form, differences in Poisson’s ratio between self-sensing sensors and host concrete also play significant roles in piezoresistive performance. Given these considerations, the enhanced sensitivity of self-sensing cement-stabilized sand under loading condition d (loading on top of electrodes), shown in Figure 7, can be attributed to changes in Poisson’s ratio originating from alterations in the dimensions of electrodes and self-sensing cement-stabilized sand. However, the influence of Poisson’s ratio on the self-sensing functionality of cement-stabilized sand requires detailed exploration in future research endeavors.

The findings indicate the importance of the electrode layout for successfully applying self-sensing cement-stabilized sand. Considering the loading region, if the electrode position is not selected mindfully, the stress-sensing capability of self-sensing cement-stabilized sand, particularly under low stress levels, will be insignificant. Therefore, when self-sensing cement-stabilized sand is used in bulk form, the distance and number of electrodes used to collect electrical resistance data play crucial roles in successfully applying this intelligent material for stress-sensing purposes. Given this issue, the sensitivity of self-sensing cementitious composites has been widely investigated in the literature by installing electrodes directly under stress regions in the laboratory [47,49,84,85,86,87] and at field scales [20,88,89,90]. The results obtained from the current study regarding the influence of loading position on the piezoresistive performance of self-sensing cementitious composites have been neglected in previous studies; thus, the outputs presented in this study are novel [12,53,91]. The stress sensitivity of self-sensing cement-stabilized sand is considerable when electrodes are directly subjected to stress compared to the cases in which electrodes are located far from the stress regions. Therefore, it is concluded that the sensitivity of the self-sensing cementitious composite is highly dependent on the location of the electrodes and the loading region.

### 3.4. Effects of Stress Level on Piezoresistive Performance

**Figure 8:**
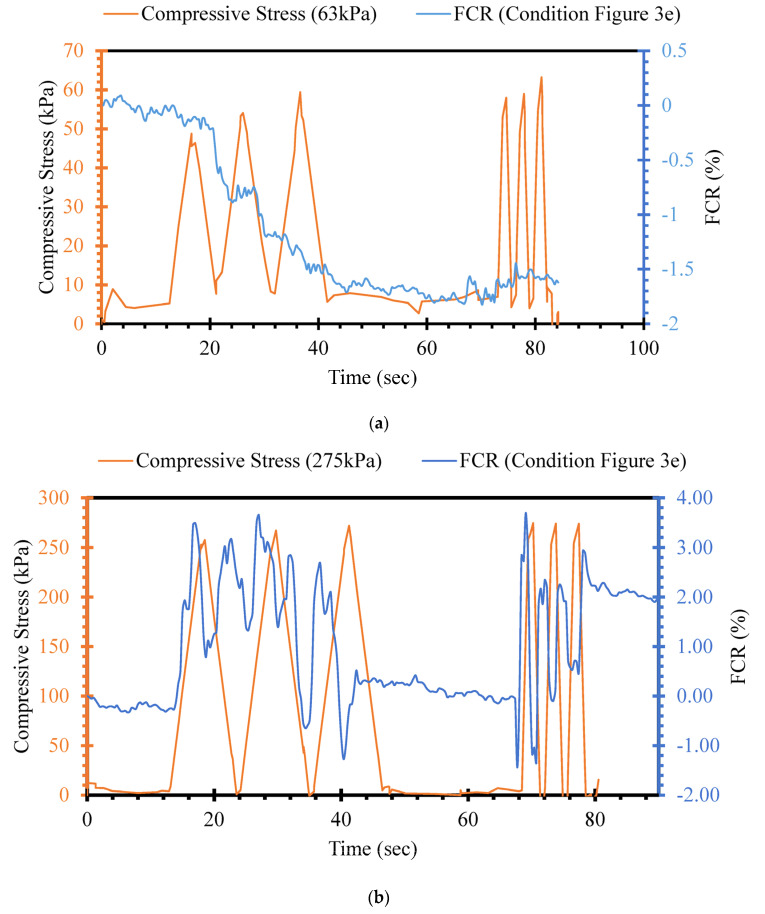

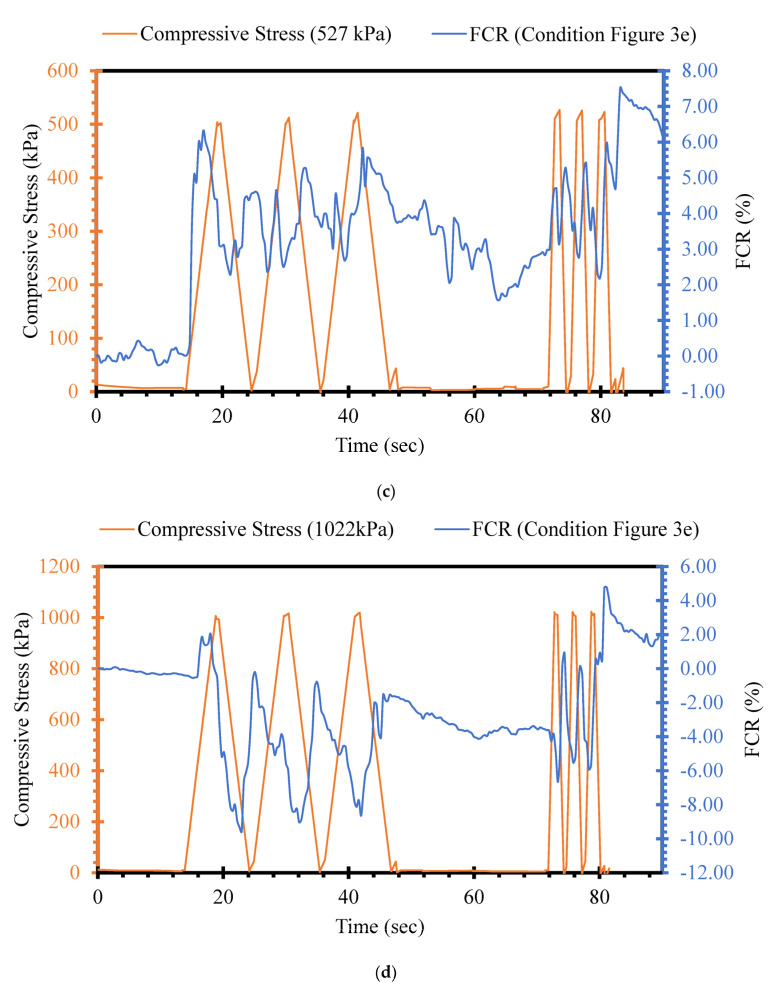
Compressive cyclic stress/FCR–time for loading conditions under 1000 kPa according to Figure 3e. (**a**) cyclic compressive stress = 63 kPa; (**b**) cyclic compressive stress = 275 kPa; (**c**) cyclic compressive stress = 527 kPa; (**d**) cyclic compressive stress = 1022 kPa.

**Figure 9:**
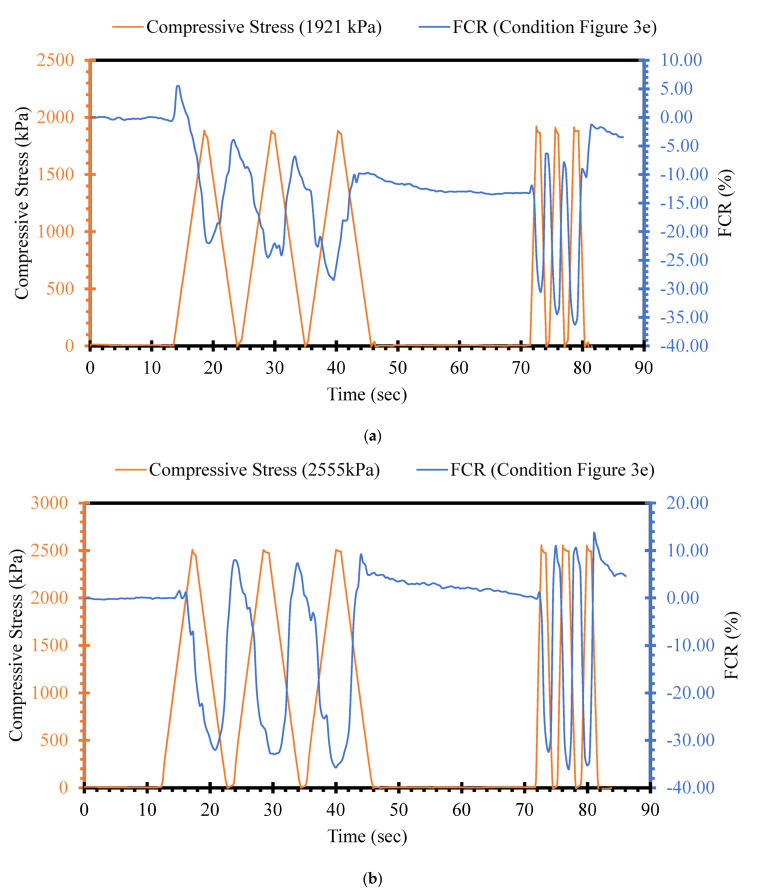

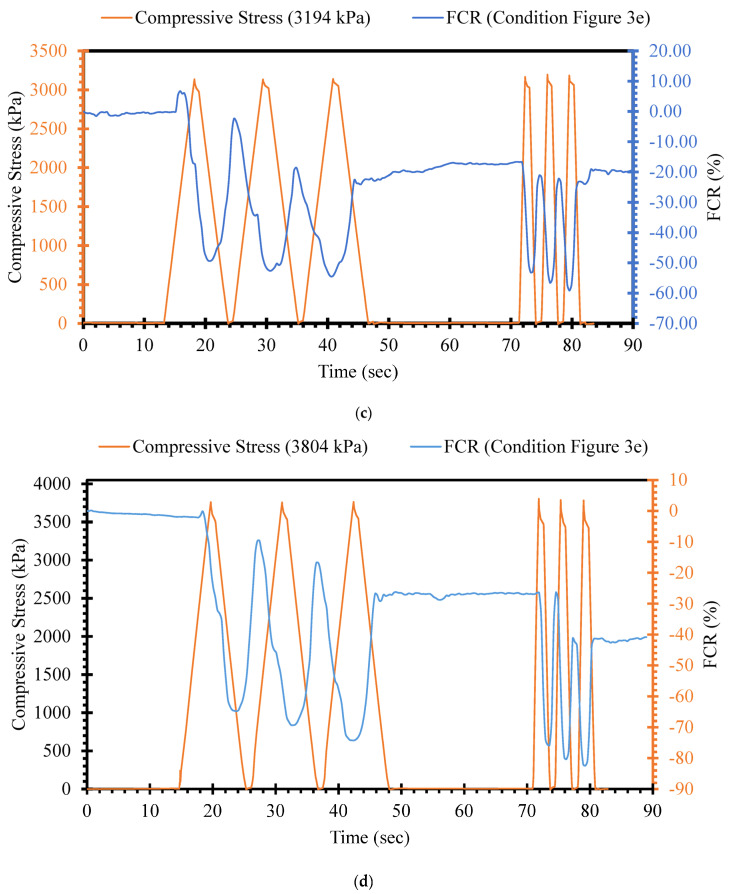
Compressive cyclic stress/FCR–time for loading conditions over 1000 kPa according to Figure 3e. (**a**) cyclic compressive stress = 1921 kPa; (**b**) cyclic compressive stress = 2555 kPa; (**c**) cyclic compressive stress = 3194 kPa; (**d**) 3804 kPa.

Among other factors, the stress level induced by the applied external load is one of the main factors affecting the piezoresistive performance of self-sensing cementitious composites [92,93,94]. In this section, the FCR changes in self-sensing cement-stabilized sand containing 4% MWCNT/GNP were evaluated under the loading conditions shown in Figure 3e by applying various stress levels ranging from 63 kPa to 3804 kPa, as illustrated in Figure 8 and Figure 9. Figure 8 and Figure 9 illustrate the incremental sensing ability change with increasing stress levels. For instance, as seen in Figure 8a, the FCR change trend is unclear under 63 kPa compressive cyclic stress, indicating that the cement-stabilized sand containing 4% MWCNT/GNP lacks the sensitivity to detect changes in the transportation infrastructure field below this stress level. However, a sudden change in FCR is seen in Figure 8a after the first compressive cyclic stress, showing the negligible stress-sensing capability of the material. Although the FCR change trends started following the compressive cyclic stress trend after applying 275 kPa and 527 kPa, this is not evident in Figure 8b,c. The FCR changes yielded an explicit alteration pattern under 1022 kPa compressive cyclic stress, as seen in Figure 8d.

Similarly, the FCR variations continued to increase with increasing stress level, as shown in Figure 9. The findings in Figure 8 and Figure 9 indicate the considerable effects of stress levels on the sensing capability of self-sensing cement-stabilized sand. Self-sensing cement-stabilized sand can be used in field situations for strain, damage, and traffic detection. This detection, performed using self-sensing cementitious composites, would not be possible for low stress levels. However, the damage could be detected by considering the initial and sudden changes in the FCR trend under compressive cyclic stress, even in the case of a small level of stress. Previous studies provided comprehensive information regarding damage detection under cyclic and monotonic loading [55,95]. In addition, it should be noted that the strain-sensing capability of self-sensing cement-stabilized sand is highly dependent on the strain level caused by applied stress. The strain increases with increasing stress levels; hence, the FCR changes become clearer with increasing strain levels. Given this issue, it is clear in Figure 8 and Figure 9 that the FCR decreases during the loading stages and increases during the unloading stages. The loading stages cause the shrinkage of voids and decreased distances between functional fillers, leading to decreasing FCR. The larger the stress level, the more considerable the decrease in FCR will be due to compressed voids and emerging electrically conductive pathways. In general, the results achieved regarding the influence of stress level on the piezoresistive performance of self-sensing cementitious composite in this study are coherent with those of previous studies [23,64,96].

This section discusses the correlation between FCR and stresses for load conditions applied to the small surface of prismatic specimens, as illustrated in Figure 3e. FCR is defined as follows.

(2) $$FCR=\frac{\Delta R}{R_{0}}\times 100={f(\sigma }_{x})$$

**Figure 10:**
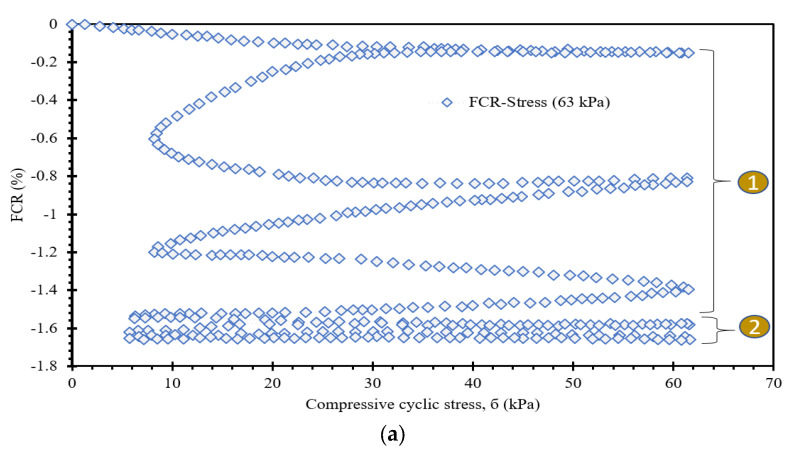

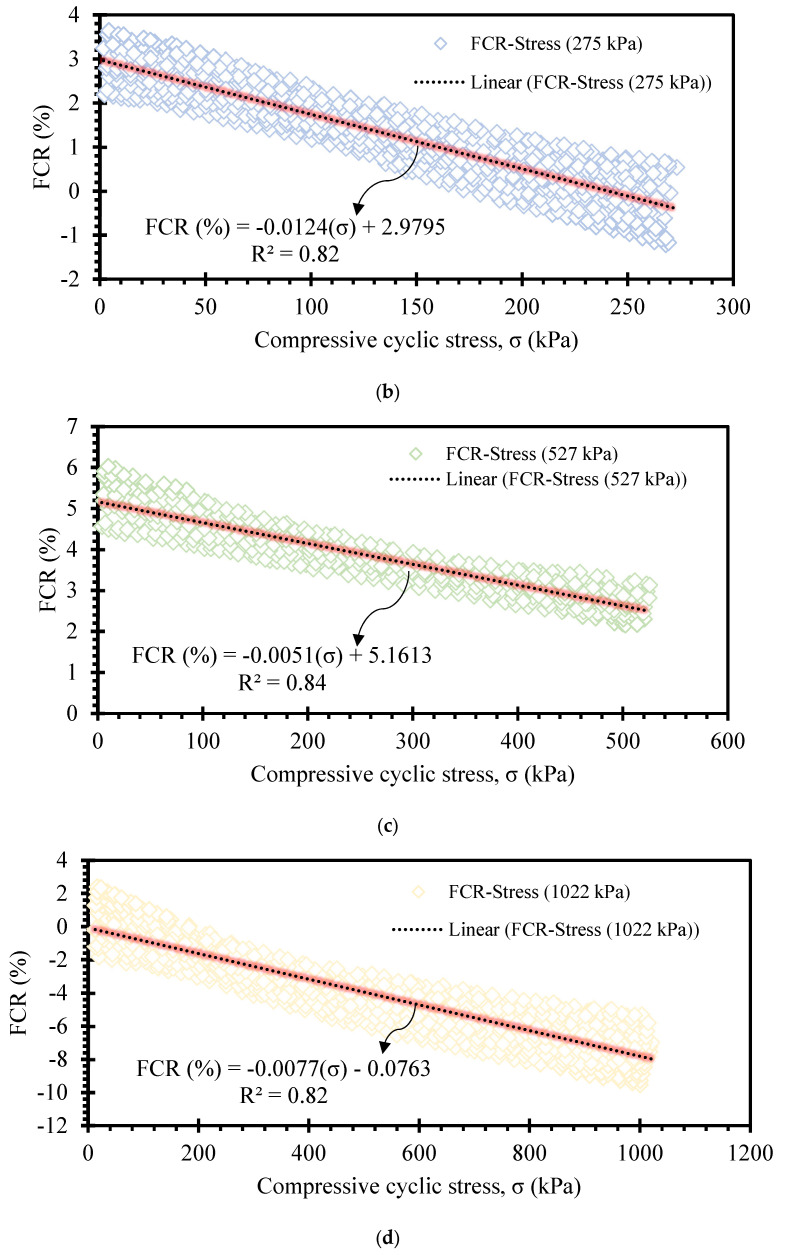
FCR-Stress relationship. (**a**) cyclic compressive stress = 63 kPa; (**b**) cyclic compressive stress = 275 kPa; (**c**) cyclic compressive stress = 527 kPa; (**d**) cyclic compressive stress = 1022 kPa.

**Figure 11:**
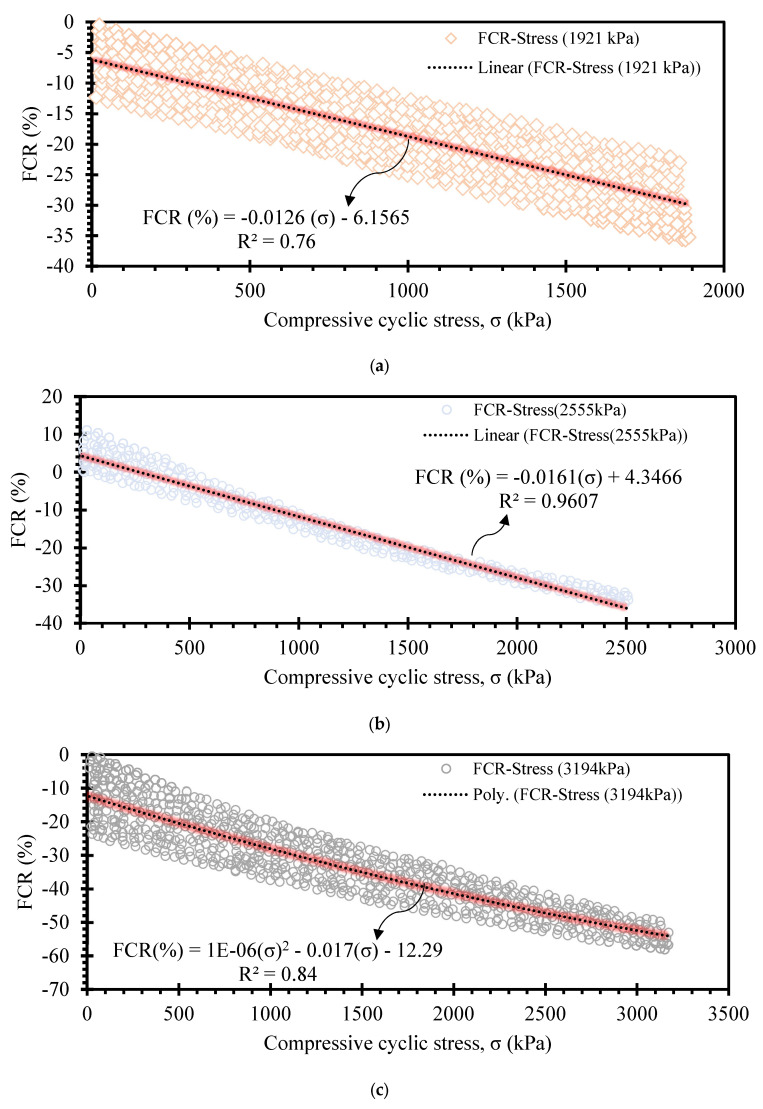

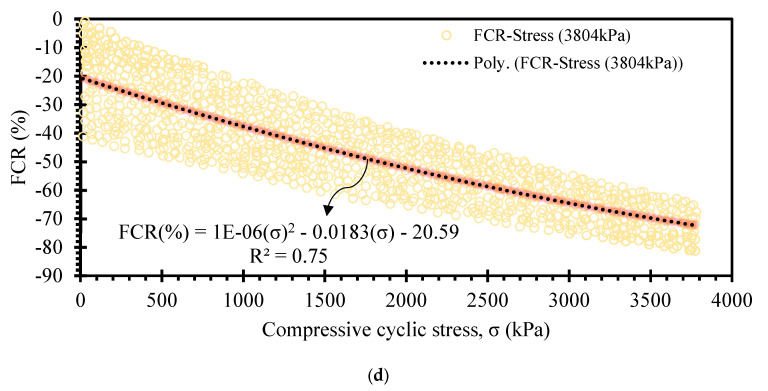
FCR-Stress relationship. (**a**) cyclic compressive stress = 1921 kPa; (**b**) cyclic compressive stress = 2555 kPa; (**c**) cyclic compressive stress = 3194 kPa; (**d**) 3804 kPa.

As discussed, the FCR decreases and increases under cyclic compressive loading and unloading stages, respectively. However, hysteresis in FCR may occur due to various factors, including the type of components of the self-sensing cementitious composite, temperature variation, and stress level. The FCR changes are highly dependent on the induced stress level, as seen in Figure 10 and Figure 11. The self-sensing cementitious composite may not be sensitive under a small stress level, as seen in Figure 10a for 63 kPa cyclic compressive stress. In Figure 10a, two separate regions of FCR changes under cyclic compressive stress are evident. In the first region, sudden changes in FCR occurred under the first three stress cycles, which can also be observed in Figure 8a. In the second region, the change in FCR under cyclic compressive stress was negligible. The drastic changes in FCR in the first region are attributed to the initial accommodation of the cementitious composite and compression of the interface between the electrodes and smart material. After this phase, the stress sensitivity of self-sensing cement-stabilized sand under 63 kPa becomes almost zero in the second region in Figure 10a. This phenomenon can also be observed in Figure 8a, in which the FCR drastically decreases under the first three stress cycles and becomes almost constant under subsequent cycles.

The stress sensitivity of self-sensing cement-stabilized sand started to rise upon increasing the stress level, as seen in Figure 10b–d and Figure 11a–d. Regarding Figure 10b–d and Figure 11a–d, it is observed that the FCR changes increase with increasing stress levels. The increasing trend in FCR changes under increasing compressive cyclic stress indicates enhanced sensitivity performance. However, reversibility is another factor that should be considered in self-sensing cementitious composites. The findings indicate that the self-sensing cement-stabilized sand could not sense the small stress level. Conversely, a linear correlation was achieved between FCR changes and stress levels up to 2555 kPa (approximately 67% of ultimate strength). Beyond 67% of ultimate strength, a polynomial correlation was established between FCR changes and compressive cyclic stress, as exhibited in Figure 11c,d. The linear correlation between the FCR and compressive cyclic stress indicates the reversibility of FCR and the irreversibility of baseline electrical resistance. This issue can be observed in Figure 8 and Figure 9, where the FCR is almost completely reversible after each loading and unloading under compressive cyclic stress, reaching up to 2555 kPa, while the baseline resistance is not reversible. Beyond 2555 kPa, both FCR changes and baseline resistance yielded an irreversible trend, as seen in Figure 9c,d. In previous studies, it was found that FCR is reversible under cyclic compressive stress up to 75% of ultimate strength, but that it would be irreversible were the cyclic compressive stress to exceed 75% of ultimate strength, indicating that the findings are coherent with previous study results [97]. It should be noted that the most considerable repeatability was observed under 2555 kPa cyclic compressive stress, as seen in Figure 9b and Figure 11b.

### 3.5. Microstructure Analysis

**Figure 12:**
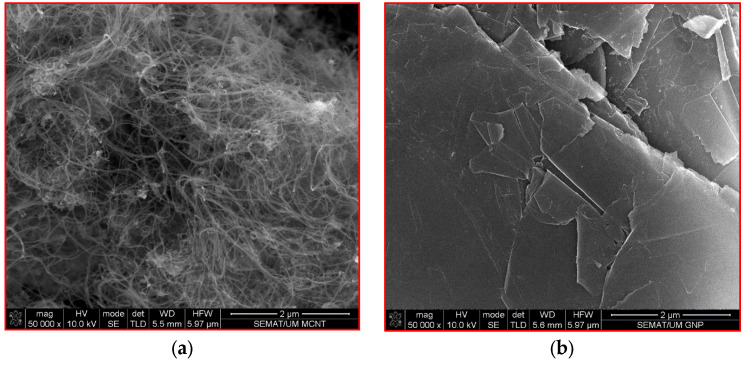
Morphology of (**a**) MWCNTs and (**b**) GNPs.

The morphologies of the MWCNTs and graphene nanoplatelets are illustrated in Figure 12a and Figure 12b, respectively. MWCNTs composed of buckytubes are two-dimensional and form a hollow structure. Figure 12a indicates a high aspect ratio (length/diameter) for MWCNTs; thus, this high aspect ratio results in bridging effects in cementitious composites. The bridging effects of MWCNTs increase the electrical conductivity between cementitious composites and enhance mechanical strength [98]. Figure 12b presents the microstructure of the graphene nanoplatelets (GNP) utilized in this study. The GNPs are lightweight and have a low density, excellent mechanical characteristics, high specific surface area, and electrical conduction characteristics. Given these features, adding GNPs to cement-stabilized sand increases the electrical conductivity and mechanical strength.

**Figure 13:**
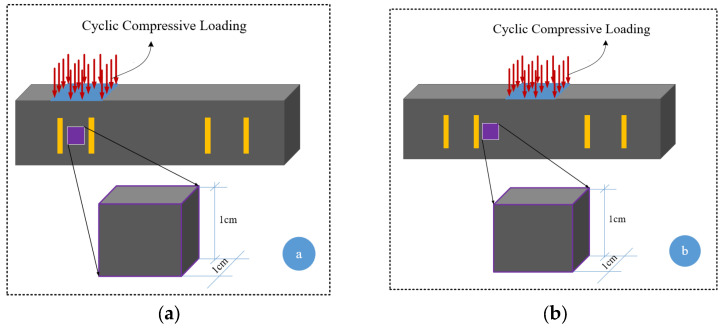
Location of sampling for microstructural analysis after electromechanical testing. (**a**) Region under cyclic compressive loading; (**b**) Region outside cyclic compressive loading.

As discussed earlier, the piezoresistive performance of the self-sensing cementitious composite depends on loading conditions based on the electrode location used for recording the electrical resistance. To analyze this issue according to the microstructure condition using SEM analysis, 1 cm^3^ samples were provided from two locations under the loading region and outside of the loading region, as seen in Figure 13a,b. After electromechanical testing, the first sample shown in Figure 13a was prepared from the region directly subjected to loading. In contrast, the sample shown in Figure 13b was provided from the location outside of the loading region.

**Figure 14:**
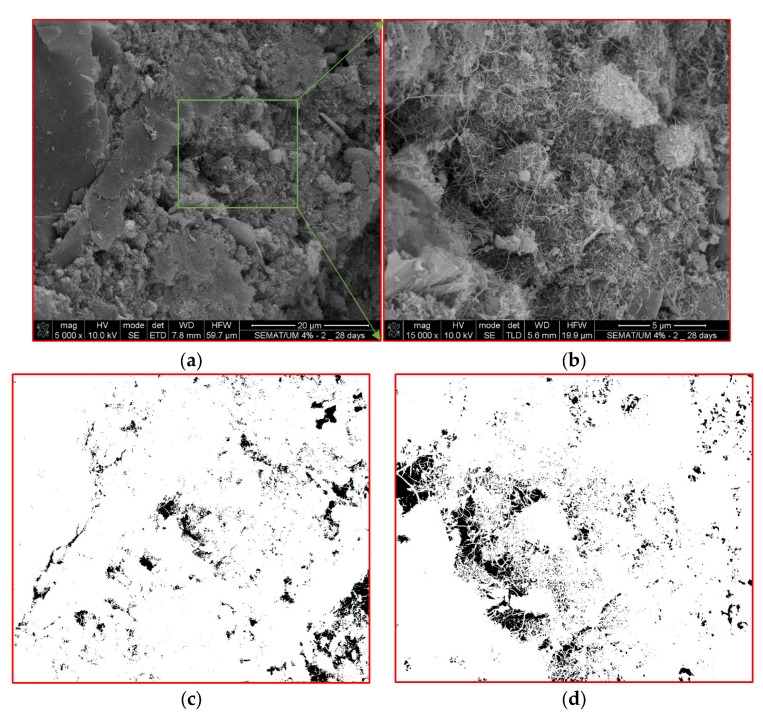
Microstructure of self-sensing cementitious composite sampled from the under-loading region according to Figure 12a. (**a**) Scale = 20 µm; (**b**) Scale = 5 µm; (**c**) Binary image of Figure 14a; (**d**) Binary image of Figure 14b.

**Figure 15:**
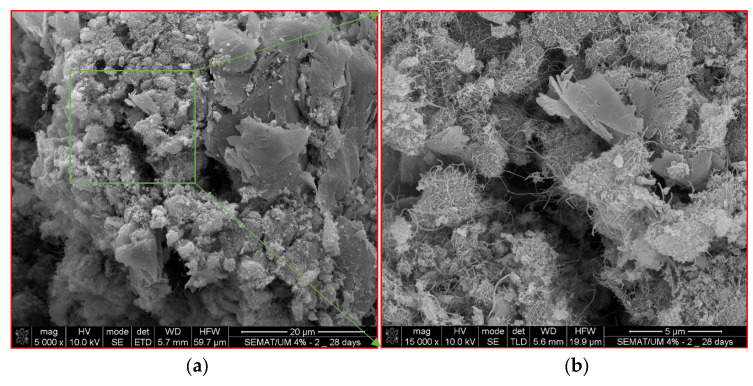

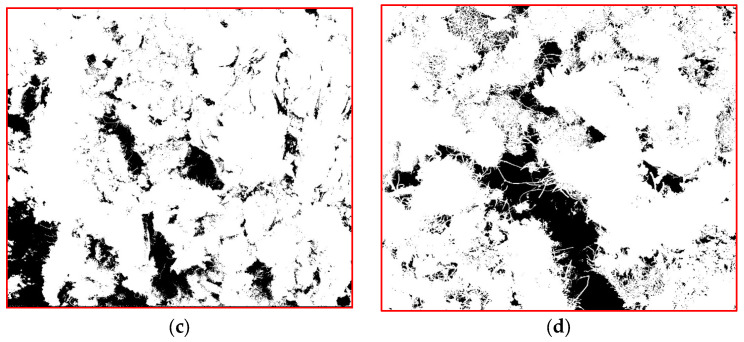
Microstructure of self-sensing cementitious composite sampled from outside the loading region according to Figure 12b. (**a**) Scale = 20 µm; (**b**) Scale = 5 µm; (**c**) Binary image of Figure 15a; (**d**) Binary image of Figure 15b.

The existing pores in the self-sensing cementitious composite represent one of the main influential factors affecting piezoresistive performance [99]. Thus, the variation in electrical resistance with regard to loading conditions can be clearly explained through SEM morphologies. The microstructural features of the sample prepared from the region under loading (see Figure 12a) are shown in Figure 14. On the other hand, the microstructural features of the sample taken from the region outside of the loading area (see Figure 13b) are presented in Figure 15. The pores and voids shown in black spots in Figure 14 are smaller than those in Figure 15. Therefore, comparing Figure 14 and Figure 15 indicates the densified microstructures for the sample provided from the region under loading conditions (Figure 14) compared to the sample taken from a region outside the loading area (Figure 15). The higher stress sensitivity of self-sensing cement-stabilized sand in the loading condition on top of electrodes could be due to the compacted and densified microstructure that emerged upon loading. Given this issue, when the electrical resistance is recorded from electrodes covered by the loading area, the piezoresistive performance of self-sensing is considerable compared to the case in which the electrical resistance is recorded using the electrodes outside of the loading area.

The cement hydration and pozzolanic reactions produce calcium silicate hydrate (CSH) and calcium aluminate hydrate (CAH), leading to strength gains in calcium-based stabilized geomaterials [100,101], particularly cement-stabilized composites [102,103]. A previous study’s findings showed that cement hydration products fully adhere to the surface of carbon nanomaterials [104]. The adhesion of cement hydration products to the surface of carbon nanomaterials is due to their considerably large specific surface area and high surface energy [105]. Given this issue, the MWCNT/GNP bridges the pores and cement hydration products, resulting in conductive pathways. In the portion of the sample subjected directly to loading, the bridging effects of MWCNT/GNP accumulate further, as seen in Figure 14. In contrast, the bridging effects of MWCNT/GNP outside the loading region remain unchanged or decrease due to tension, as can be observed in Figure 15. Therefore, resistance variation occurs in the self-sensing cement-stabilized samples, resulting in different sensitivity responses to the loading conditions.

**Figure 16:**
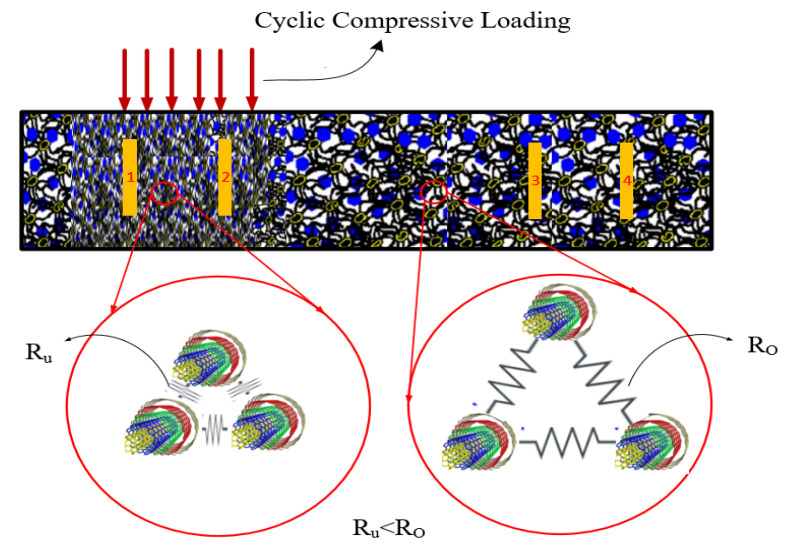
Schematic illustration of the microstructure of the self-sensing cementitious composite of the under-loading region (R_u_) and out-of-loading region (R_o_).

To further analyze the effects of microstructures on the sensitivity of cement-stabilized sand with regard to loading conditions, a schematic illustration shown in Figure 16 is considered. Figure 16 exhibits a schematic illustration of the microstructure condition under loading on top of two electrodes (electrodes 1 and 2). The micro- and nanovoids decrease under compressive loading. In contrast, the effects of loading on the microstructure compression decrease with increasing distance from the loading area, as shown in Figure 16. Given this phenomenon, when the specimen is loaded onto a specific surface, the resistance of self-sensing cement-stabilized sand under loading is not homogeneous throughout all samples. Depending on the location of the loading area, the electrical resistance differs. For instance, the electrical resistance of the portion directly subjected to loading (R_u_) is smaller than that of regions far from the loading area (R_o_), as illustrated in Figure 16. Therefore, the piezoresistive performance of self-sensing cement-stabilized sand substantially depends on the electrode location used to record electrical resistance. For instance, considering the two-electrode probe system circuit, the electrical resistance in Figure 16 could be recorded by using different connection options between electrodes (i.e., 1–2; 1–3; 1–4; 2–3; and 2–4 options). Regarding Figure 16, the piezoresistive performance of self-sensing cement-stabilized sand can be considerable if the electrical resistance is recorded using electrodes 1 and 2 (1–2 option) compared to cases in which the electrical resistance is recorded using other electrode layout options (1–3; 1–4; 2–3; and 2–4). This issue is attributed to the densified and loose microstructures occurring under the loading region and outside of the loading regions, respectively, as seen in Figure 14 and Figure 15.

While a brief examination of microstructure analysis has been undertaken, additional investigations are necessary to thoroughly delve into the impact of loading conditions on the microstructural alterations of self-sensing cement-stabilized sand, employing both experimental and numerical approaches.

## 4. Conclusions

The influence of various cyclic compressive loading conditions on the electromechanical performance of self-sensing cement-stabilized sand was evaluated. In addition, SEM was conducted to explore the effects of loading conditions on the morphological features of self-sensing cement-stabilized sand. Based on the experimental findings, the following conclusions are drawn:

1. The distance between electrodes used for electrical resistance recording considerably affects sensitivity.
2. The distance of the loading region from the electrodes employed for electrical resistance recording considerably affects the electromechanical performance of cement-stabilized sand.
3. Depending on the location of electrodes relative to the loading region, the self-sensing cement-stabilized sand yielded various performances under the same stress level. The best sensitivity was observed when the electrodes were located directly under the loading region.
4. The FCR increased with increasing stress level, showing the enhanced sensitivity of self-sensing cement-stabilized sand with increasing stress levels. However, the reversibility decreased when the applied stress level was more than 67% of the ultimate strength of cement-stabilized sand.
5. The FCR suddenly decreased under the few cycles of the applied low stress level (63 kPa), and then it became constant under subsequent cycles of the same stress level. This issue can be attributed to the effects of accommodation that occurred under the first few cycles. Therefore, the accommodation effects at the beginning of loading must be considered in order to calibrate self-sensing cement-stabilized sand performance.
6. The SEM results showed the accumulated bridging effects of carbon nanomaterials under the loading region and the weakened bridging effects outside the loading region. Therefore, the considerable sensitivity of self-sensing cement-stabilized sand under the condition of loading directly on top of electrodes may be due to the accumulated bridging effects of carbon nonmaterial, which could provide random conductive networks.

The findings present the effects of the loading conditions and level on the electromechanical performance of self-sensing cement-stabilized sand. The findings indicate the crucial role of electrode layout in the sensing performance of cement-stabilized sand, something which needs to be considered before applying this smart material in civil engineering projects.

## Figures and Tables

**Table 1:** Physical properties of standard sand.

Mesh Size (mm)	0.08	0.16	0.5	1	1.6	2
Cumulative retained (%)	99 ± 5	87 ± 5	67 ± 5	33 ± 5	7 ± 5	0
Specific gravity	2.67	Uniformity coefficient = 7.5			Curvature coefficient = 1.8	

**Table 2:** Chemical and physical properties of CEM 1, 42.5R.

SiO_2_	Al_2_O_3_	Fe_2_O_3_	MgO	CaO	Na_2_O	TiO_2_	K_2_O	MnO	P_2_O_5_	SO_3_
19.94	4.76	3.38	1.31	63.93	0.17	0.24	0.44	0.075	0.063	2.54
Loss on Ignition (LOI)			Fineness (m^2^/kg)				Specific Gravity			
2.97			360				3.15			

**Table 3:** Characteristics of GNPs and MWCNTs at 0 °C.

GNP										
Surface Area (m^2^.g^−1^)	Density (g/cm^3^)	Carbon Content (%)	Tensile Modulus (GPa)	PH Value (30 °C)	Tensile Strength (GPa)	Layers	Dimension		Form	Part Number
120–150	0.6	>99.5	1000	7–7.65	5	10< *n* <60	Thickness	Diameter	Gray Powder	TGN201
							4–60 nm	5–10 µm		
MWCNT										
Surface Area (m^2^.g^−1^)	Density (g/cm^3^)	Color	Outside Diameter (nm)	Length (µm)	Ash (wt.%)		Carbon Content (%)		Part Number	
350	0.27	Black	<50	10–30	<1.5		>98		GCM327	

**Table 4:** Components of self-sensing cement-stabilized sand.

CNMs (%)	MDD (kg/m^3^)	Sand (kg/m^3^)	Cement (kg/m^3^)	Water (kg/m^3^)	CNMs (kg/m^3^)	Pluronic F-127 (kg/m^3^)	TBP-97% (kg/m^3^)
0.5	2120	1806.12	180.61	122.88	9.03	0.90	0.45
1	2080	1735.57	173.56	150.92	17.36	1.74	0.87
2	1970	1580.30	158.03	195.32	31.61	3.16	1.58
3	1860	1433.76	143.38	233.40	43.01	4.30	2.15
4	1800	1355.24	135.52	256.54	54.21	5.42	2.71

## Data Availability

The datasets used or analyzed during the current study are available from the corresponding author on reasonable request.
